# Efficacy and safety of Tian moxibustion in treating allergic rhinitis: A protocol for a systematic review and meta-analysis

**DOI:** 10.1097/MD.0000000000023848

**Published:** 2020-12-18

**Authors:** Gen Deng, Qun Wan, Jinlong Wang, Wenguo Ye

**Affiliations:** a College of Acupuncture and Massage, Jiangxi University of Traditional Chinese Medicine; b Affiliated Hospital of Jiangxi University of Traditional Chinese Medicine, Nanchang, China.

**Keywords:** allergic rhinitis, moxibustion therapy, systematic review and meta-analysis, Tian moxibustion

## Abstract

**Background:**

Allergic rhinitis, abbreviated AR, modern medicine considers AR to be a chronic inflammatory reactive disease of the nasal mucosa mediated by exposure to allergens such as pollen and mites immunoglobulin E. AR not only affects patients’ daily life, sleep, work, and study, but also brings huge economic burden to patients and society. At present, desensitization therapy, antiallergic drugs, antihistamines, hormones, and other drugs are used to improve symptoms or immune regulation, but the clinical short-term and long-term efficacy is general, the symptoms are easy to be repeated after drug withdrawal, and the long-term toxicity and side effects of drugs are obviously insufficient. Tian moxibustion therapy has a good effect on AR. Therefore, this paper will carry out a systematic evaluation and meta-analysis of the efficacy and safety of moxibustion in the treatment of allergic rhinitis.

**Methods:**

Eight electronic databases will be searched, including PubMed, Embase, Web of Science, Cochrane Library, the China National Knowledge Infrastructure (CNKI), Chinese Science and Technology Periodical Database (VIP), Wanfang Database (WF), and Chinese Biomedical Literature Database (CBM). We will search above electronic databases from the beginning to November 2020, without any language restriction, but involving only the human subjects. Clinical efficacy, including total effective rate or cure rate, and recurrence rate will be accepted as the primary outcomes. The Rhinoconjunctivitis quality of life questionaire (RQLQ) score, symptom score (nasal congestion, snot, continuous sneezing) will be used as secondary outcomes. The Cochrane Handbook of Systematic Review (5.3.0) randomized controlled trials (RCT) risk assessment tool will be used to evaluate the risk of bias by 2 independent researchers.

**Results:**

After the completion of this study, the results will be reported, so it is not possible to give accurate results at present.

**Conclusions:**

The results of this study will provide reliable evidence for the efficacy and safety of Tian moxibustion in the treatment of allergic rhinitis.

**Ethics and dissemination:**

This paper does not need to be approved by the Ethics Committee, because this paper is a systematic review and quality evaluation of relevant literature. The results of this study will be disseminated in the form of a paper to help better guide the clinical practice of Tian moxibustion in the treatment of allergic rhinitis.

**INPLASY Registration number:**

INPLASY2020110058.

## 1 Introduction

Allergic rhinitis, abbreviated AR, modern medicine considers AR to be a chronic inflammatory reactive disease of the nasal mucosa mediated by exposure to allergens such as pollen and mites immunoglobulin E.^[1]^ Clinical symptoms are nasal congestion, runny nose, itching nose, sneezing, or accompanied by eye itching, eye red, tears, and other symptoms.^[2]^ The survey shows that the prevalence of AR in the world is as high as 10% to 20%. An estimated 600 million people worldwide suffer from AR, and the incidence is increasing.^[3]^ AR recurrent attacks may induce many respiratory complications such as allergic sinusitis nasal polyps, asthma, and chronic bronchitis, severe can be combined with asthma, otitis media, and sleep disorders.^[4]^ It not only affects the daily life, sleep, work, and study of patients, but also brings huge economic burden to patients and society.^[5]^ At present, modern medicine with desensitization therapy, the use of anti-allergic drugs, antihistamines, hormones, and other improved symptoms or immune regulation, but clinical near and long-term efficacy is general, after stopping the symptoms are easy to repeat, long-term drug toxicity side effects are obvious.^[6]^ According to the theory of viscera meridian and the thought of the unity of nature and man, the basic therapy is divided into internal treatment and external treatment.^[7]^ Tian moxibustion is a traditional Chinese medicine treatment of diseases external treatment, the use of skin irritation drugs applied to acupoints or affected areas, so that the local skin naturally congested, flushed, or blister treatment. It is found that Tian moxibustion therapy has a good effect on AR.^[8–12]^ However, there is still a lack of systematic evaluation on the efficacy and safety of Tian moxibustion therapy for AR in clinical practice. Therefore, the effectiveness and safety of Tian moxibustion in the treatment of AR will be systematically evaluated and meta-analyzed in this paper.

## 2 Methods

### 2.1 Inclusion criteria for study selection

#### 2.1.1 Types of studies

Clinical randomized controlled trials (RCTs) containing Tian moxibustion for AR were included, with no limitation of language and publication status.

#### 2.1.2 Types of participants

There are clear and recognized diagnostic criteria and efficacy criteria, and all patients are diagnosed as AR, regardless of sex, age, and origin of the case.

#### 2.1.3 Types of interventions

##### 2.1.3.1 Experimental interventions

Tian moxibustion therapy, or mixed therapies based on Tian moxibustion will also be include.

##### 2.1.3.2 Control interventions

The control group will receive one of the following treatment methods: conventional pharma-cological therapy, no treatment, and placebo.

#### 2.1.4 Types of outcome measures

##### 2.1.4.1 Primary outcome

Clinical efficacy, including total effective rate or cure rate, and recurrence rate will be accepted as the primary outcomes.

##### 2.1.4.2 Secondary outcomes

The Rhinoconjunctivitis quality of life questionaire (RQLQ) score, symptom score (nasal congestion, snot, continuous sneezing) will be used as secondary outcomes.

### 2.2 Exclusion criteria

Non-randomized controlled trials; no exact diagnostic scale or therapeutic scale; no Tian moxibustion as the main treatment in the experimental group, and Tian moxibustion therapy was found in the control group. Repeated literature; theory and review literature; animal experiments; nursing research.

### 2.3 The retrieval methods and strategies of this study

#### 2.3.1 Electronic database retrieval

Eight electronic databases will be searched, including PubMed, Embase, Web of Science, Cochrane Library, the China National Knowledge Infrastructure (CNKI), Chinese Science and Technology Periodical Database (VIP), Wanfang Database (WF), and Chinese Biomedical Literature Database (CBM). We will search above electronic databases from the beginning to November 2020, without any language restriction, but involving only the human subjects. According to the principle of Participants,Intervention,Control, we will search the relevant literature by combining subject words with free words, search terms consist of disease (“allergic rhinitis” or “allergic rhinitides” or “rhinitides, allergic”) and intervention (“tian moxibustion” or “acupoint application” or “foaming moxibustion” or “crude herb moxibustion” or “Vesiculation”) and research types (randomized controlled trial or controlled clinical trial or random trials or RCT). The PubMed search strategy is shown in Table 1.

**Table 1:** Retrieval strategies in PubMed.

ID	Query
#1	“Allergic Rhinitis”[Mesh]
#2	(((“Allergic Rhinitides”[Title/Abstract]) OR (“Rhinitides, Allergic”[Title/Abstract])) OR (“Rhinallergosis”[Title/Abstract])) OR (“Hypersensitive Rhinitis”[Title/Abstract])
#3	#1 OR #2
#4	(((“Tian Moxibustion”[Title/Abstract]) OR (“Acupoint Application”[Title/Abstract])) OR (“Foaming Moxibustion”[Title/Abstract])) OR (“Vesiculation”[Title/Abstract])
#5	(((“Randomized Controlled Trial”[Title/Abstract]) OR (“Randomized Clinical Trial”[Title/Abstract])) OR (“Randomized Trial”[Title/Abstract])) OR (“Rct”[Title/Abstract])
#6	#3 AND #4 AND #5

#### 2.3.2 Searching other resources

This study will combine manual retrieval of literature resource database to search relevant conference papers that meet the inclusion criteria. In addition, the grey literature, as well as ongoing and recently completed studies, will be searched on Clinicaltrials.gov.

### 2.4 Data extraction and management

#### 2.4.1 Literature inclusion and data extraction

**Figure 1:**
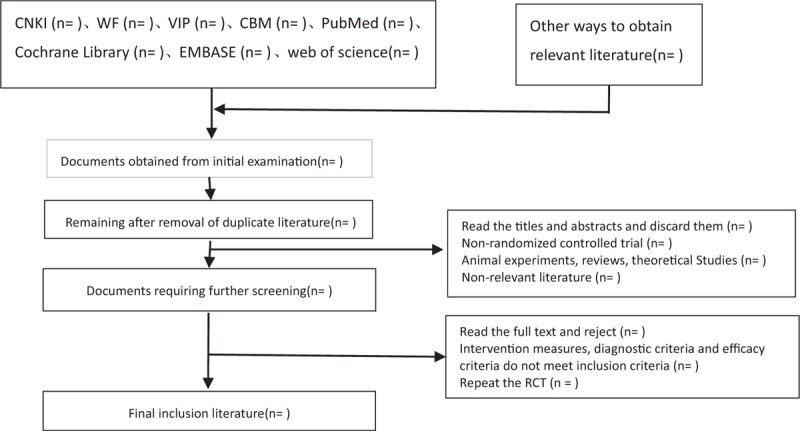
Flow chart of literature incorporation. (This flowchart is about the inclusion and exclusion of the literature in order to obtain the final document).

The 2 researchers independently read the title and abstract of the literature we obtained, read the full text of the trials that might meet the inclusion criteria to determine whether the inclusion criteria were truly met, and discussed the conflicting literatures or let the third researcher decide whether to include them. Two researchers independently extracted data from the included studies, including study design, intervention measures and methods, measurement indicators, results, methodological contents such as hidden grouping and blind method, etc, and a third evaluator checked the consistency of the data. If the required information is incomplete, we will contact the original author for the required data. The inclusion process of this study will be carried out as shown in Fig. 1.

#### 2.4.2 Methodological quality evaluation

The Cochrane Handbook of Systematic Review (5.3.0) RCT risk assessment tool will be used to evaluate the risk of bias by 2 independent researchers, including: the method of random sequence generation; allocation hiding; whether the subjects and the implementer of the treatment plan should be blinded; blind method shall be applied to evaluators; integrity of result data; selective reporting of results; other bias. According to the results of each study that meets the inclusion criteria, according to the above 7 items, objectively judge that each study is high-risk or low-risk or unclear (no relevant information or uncertainty of bias is mentioned in the literature) and explain the reasons. If there are any differences in the above quality evaluation and data extraction process, 2 people shall discuss and resolve the differences or consult the third reviewer to deal with the differences.

### 2.5 Statistical analysis

#### 2.5.1 Quantitative data synthesis

RevMan5.3 software will be used for statistical analysis. The odds ratio (OR) and its 95% confidence interval (CI) will be used as the counting data, while the weighted mean difference (WMD) and its 95% CI will be used as the measurement data.

#### 2.5.2 Assessment of heterogeneity

The heterogeneity test will be carried out first among all studies, *I*^2^ test will be used. When *P* > .1 and *I*^2^ < 50%, the fixed effect model will be used; otherwise, the random effect model will be used. When the clinical heterogeneity between the 2 studies is large, only descriptive analysis will be performed.

#### 2.5.3 Publication bias

RevMan5.3 statistical software will be used to conduct qualitative analysis of publication bias in inverted funnel plots. If the funnel plot is asymmetric, there may be a publication bias in the research results.

#### 2.5.4 Subgroup analysis

If significant heterogeneity is found in our systematic review and sufficient data are available, we will conduct a subgroup analysis based on moxibustion type, moxibustion time, treatment cycle, and outcome measurement methods in the experimental and control groups.

#### 2.5.5 Sensitivity analysis

When sufficient RCTs are available, we will conduct sensitivity analysis by excluding low-quality or high-quality studies one by one according to methodological quality, sample size, and missing data.

## 3 Discussion

Allergic rhinitis, known as “nasal edema” in Chinese medicine, is nose congestion, sneeze, flow clear nose, nose itching, nasal blockage, and so on as the main clinical symptoms of a lung disease, the etiology is mostly lung, spleen, kidney qi deficiency, healthy gas is not solid, outside the department of defense, the head experience of the evil invasion caused by wind cold.^[13]^ Tian moxibustion is a kind of external treatment of traditional Chinese medicine. It is a treatment method to apply irritating drugs to acupoints or affected areas to make local skin naturally congested, flushed, or blister. Tian moxibustion has not only the function of stimulating acupoints, but also the dual therapeutic effect of acupoints and drugs through the absorption of specific drugs in the acupoint area, can tonify spleen and invigorate lung, cultivate yuan solid, warm lung and dissipate cold, dissipate phlegm, and open orifices.^[10]^ In recent years, because of its remarkable curative effect, this treatment method has been paid more and more attention in clinic, but we have not seen the systematic evaluation of the effectiveness and safety of moxibustion in the treatment of AR. Therefore, it is necessary to systematically evaluate the treatment of AR by Tian moxibustion in this study, which can provide evidence-based medicine evidence for future clinical guidance of the treatment of AR by Tian moxibustion.

## Author contributions

**Data curation:** Gen Deng, Qun Wan.

**Formal analysis:** Gen Deng, Qun Wan.

**Investigation:** Gen Deng, Qun Wan.

**Methodology:** Qun Wan, Jinlong Wang.

**Project administration:** Gen Deng, Wenguo Ye.

**Software:** Qun Wan, Jinlong Wang.

**Supervision:** Wenguo Ye.

**Validation:** Wenguo Ye.

**Writing – original draft:** Gen Deng, Wenguo Ye.

**Writing – review & editing:** Gen Deng, Wenguo Ye.
